# Pattern of pesticide storage before pesticide self-poisoning in rural Sri Lanka

**DOI:** 10.1186/1471-2458-9-405

**Published:** 2009-11-05

**Authors:** Fahim Mohamed, Gamini Manuweera, David Gunnell, Shifa Azher, Michael Eddleston, Andrew Dawson, Flemming Konradsen

**Affiliations:** 1 South Asian Clinical Toxicology Research Collaboration, Faculty of Medicine, University of Peradeniya, Sri Lanka; 2 The Registrar of Pesticides, Government Department of Agriculture, Peradeniya, Sri Lanka; 3 Department of Social Medicine, University of Bristol, Bristol, UK; 4 Polonnaruwa General Hospital, North Central Province, Sri Lanka; 5 National Poisons Information Service - Edinburgh, Royal Infirmary of Edinburgh, and Clinical Pharmacology Unit, University of Edinburgh, UK; 6 Department of International Health, Immunology and Microbiology, University of Copenhagen, Copenhagen, Denmark

## Abstract

**Background:**

Deliberate self-poisoning with agricultural pesticides is the commonest means of suicide in rural Asia. It is mostly impulsive and facilitated by easy access to pesticides. The aim of this large observational study was to investigate the immediate source of pesticides used for self-harm to help inform suicide prevention strategies such as reducing domestic access to pesticides.

**Methods:**

The study was conducted in a district hospital serving an agricultural region of Sri Lanka. Patients who had self-poisoned with pesticides and were admitted to the adult medical wards were interviewed by study doctors following initial resuscitation to identify the source of pesticides they have ingested.

**Results:**

Of the 669 patients included in the analysis, 425 (63.5%) were male; the median age was 26 (IQR 20-36). In 511 (76%) cases, the pesticides had been stored either inside or immediately outside the house; among this group only eight patients obtained pesticides that were kept in a locked container. Ten percent (n = 67) of the patients used pesticides stored in the field while 14% (n = 91) purchased pesticides from shops within a few hours of the episode. The most common reasons for choosing the particular pesticide for self-harm were its easy accessibility (n = 311, 46%) or its popularity as a suicide agent in their village (n = 290, 43%).

**Conclusion:**

Three quarters of people who ingested pesticides in acts of self-harm used products that were available within the home or in close proximity; relatively few patients purchased the pesticide for the act. The study highlights the importance of reducing the accessibility of toxic pesticides in the domestic environment.

## Background

Deliberate self harm is a major public health problem globally with an estimated 873,000 suicides in 2002 [1] and significant social and psychological impacts on affected families. In Asia, self-poisoning with pesticides, especially in rural areas, is a common means of self-harm and is associated with a high case fatality [2]. In countries such as China, Malaysia and Sri Lanka, 60% to 90% of all suicides are due to pesticide self-poisoning and it is estimated that there are between 250,000 to 370,000 suicides from pesticide poisoning worldwide every year [3].

Self-harm is often highly impulsive. Easy access to pesticides in and around the domestic environment in rural areas is believed to have made pesticide self-poisoning a preferred means of self-harm [4,5]. Suggested approaches to reduce pesticide fatalities include phasing out the most toxic products [6,7], introducing pesticide safe storage devices in households [8-10] and teaching safer sales practices to shop keepers[11,12].

However, the development of a sound strategy to reduce access to pesticides is hampered by a lack of knowledge on how people gained access to the pesticides, where pesticides were stored immediately prior to the self-harm attempts and reasons for ingesting particular products. Studies from China [13], India [14] and Sri Lanka [15] found that 65%, 80% and 72%, respectively, of pesticides used for self-harm were obtained from the home or close proximity to home. However, the sample sizes of the above studies were relatively small, so larger studies systematically investigating the storage of pesticides prior to their use for self-poisoning are warranted to aid the development of poisoning prevention strategies.

In this large observational study of patients with pesticide self-poisoning admitted to a district hospital in Sri Lanka, we aimed to determine from where patients obtained the pesticides, the mode of purchase, the place of storage prior to self-poisoning, and why the patients choose the particular pesticide.

## Methods

This study was nested within a larger observational study of poisoning and took place in a secondary referral hospital in Polonnaruwa, Sri Lanka. The Polonnaruwa district is located in the North Central Province where paddy cultivation is predominant and pesticide use is high. The population of the study area is 358, 984 (Male 52.3%, Female 47.7%) [16]. The area is also known for a high burden of pesticide self-poisoning [17].

Our study is based on a series of consecutively admitted self-poisoning patients presenting between June 2004 and March 2006. Patients were interviewed and examined by trained study doctors who collected epidemiological and clinical data using a standard questionnaire. Patients were asked about the pesticide ingested, storage and accessibility of pesticides immediately prior to the time of self-harm, purpose of purchase of pesticides, and reason for selection of that particular pesticide for the act of self-harm. Patients were interviewed soon after admission if they were conscious and able to participate; patients unwell on admission were interviewed prior to hospital discharge. The interviews were conducted in the ward and in the patient's own language.

Data were entered into an Excel sheet and analyzed using the Statistical Program Stata version 10.1. Pearson Chi-squared and Fisher's exact tests were used for analyzing categorical variables.

Ethical approval for this study was obtained from the Ethic Review Committees of the Colombo Medical Faculty, the Sri Lanka Medical Association, the Peradeniya Medical Faculty, and the Australian National University.

## Results

During the 20 month study period, 2019 patients with a history of poisoning were admitted to the adult medical wards of the Polonnaruwa general hospital. Of the 2019 admissions, 1012 (50%) had ingested a plant, pharmaceutical or household chemical and were excluded from further analysis. A further 125 patients were excluded from the study due to unintentional, homicidal or occupational pesticide exposure (n = 79), death before an interview could be conducted (n = 36), referral to tertiary care before interview (n = 4), or being mentally unfit for interview (n = 6 patients).

Therefore, 882 patients were eligible for inclusion in the study. However, 213 interviews were not able to be completed (14 patients refused to participate and interview data were incomplete for 199 patients). Therefore, the analysis includes 669 eligible patients. The patients included in the analysis (n = 669) had lower case fatality (3.7%, 95% CI 2.4%-5.4%) compared to those eligible patients who didn't complete or participate in the study (n = 213) [(12.6%, 95% CI 8.5%-18%), (χ^2 ^= 23.2, 1 df, p = 0.00)]. Patients who didn't participate in the study were similar in age [median age was 28 (IQR 21-39 years] and gender (66% males) compared to participants [median age 26 (IQR 20-36; 425 (63.5%) male]. Forty percent (40%) of participants were full time farmers; 20% did part time farming, while 33% did another job and 7% were students. Most of the farmers were male (78%) while most of the non-farmers were female (58%).

### Accessibility to pesticide at the time of self-harm

Forty five percent of patients ingested insecticides (67% male, 33% female), 36% herbicides (62% male, 38% female) and 5% other pesticides (56% male, 44% female) while 14% ingested unknown pesticides (58% male, 42% female). The most popular insecticides were organophosphorus compounds [(69%), 67% male, 33% female] and carbamates [(23%), 63% male, 37% female] while most common herbicides were glyphosate [(27%), 58% male, 42% female] and propanil [(23%), 48% male, 52% female].

Seventy-six percent (n = 511) of the pesticides used in the self-harm incident were obtained from a storage place either inside the home (n = 248) or just outside it (n = 263), in the home garden (Table 1). Of the pesticides obtained from close to the home, only eight were kept in locked storage. Ten percent (n = 67) accessed pesticides that were stored in the field in an open place or hidden under vegetation. Fourteen percent (n = 91) patients purchased pesticides from a shop just a few hours prior to pesticide poisoning.

**Table 1:** Patterns of pesticide storage before self-harm by gender.

	**Gender**		
		
	**Male (n = 425, 63.5%)**	**Female (n = 244, 36.5%)**	**Total (n = 669) (%)**
**Stored in the field**	50 (11.8%)	17 (7.0%)	67 (10%)

**Stored inside or outside home including in the garden**	299 (70.3%)	212 (86.8%)	511 (76%)

**Obtained from a shop**	76 (17.8)%	15 (6.1%)	91 (14%)

The source of pesticides used for self-harm differed between gender (χ^2 ^= 24.8, 2 df, p = 0.000). Men were more likely to obtain the pesticide from the fields or from a shop; whereas women were more likely to obtain the pesticide from the house or home garden (Table 1). In the study participants who subsequently died in the hospital (n = 25), the sources of pesticides were not different from those who survived (n = 644) [χ^2 ^= 0.21, 2 df, p = 0.90]

There was an association between a person's involvement in farming and the source of pesticides used for self-harm [χ^2 ^= 10.50, 2 df, p = 0.005] (table 2). More farmers obtained pesticides from the fields (52/397, 13.1%) than non-farmers (15/272, 5.5%). However, similar proportions of farmers and non-farmers obtained pesticide for self-harm from in or near their home or from a shop.

**Table 2:** Association between types of job and storage of pesticides used for pesticide self-harm.

	**Farmers (n = 397, 60%)**	**Non-farmers (n = 272, 40%)**	**Total (n = 669) (%)**
Stored in the fields	52 (13.1%)	15 (5.5%)	67 (10%)

Stored inside or outside home including in the garden	295 (74.3%)	216 (79.4%)	511 (76.4%)

Obtained from a shop	50 (12.6%)	41 (15.1%)	91 (13.6%)

### Purchasing behavior

About half of the pesticides had been purchased by the patients themselves [323/669, (48%)] and half had been bought by relatives [346/669, (52%)]. Most had been bought for cultivation (533/669; 80%); 26/669 (4%) had been bought for domestic pest control. The remaining pesticides [110/669, (16%)] had been bought specifically by the patient for the act of self-harm; among this group a subset of 91 patients obtained pesticides only a few hours prior to the poisoning incident. Nineteen patients bought the pesticide for self-harm but stored it in the house for some time (1-2 days) before the act.

### Reason for the choice of pesticide for the self-harm

We asked patients about their main reason for ingesting the particular pesticide. The reasons given were: easy accessibility (311/669 patients, 46%); popular suicide agent in their village (290/669, 43%); quick action on pests (25/669, 4%), pesticide color (whether the colour of pesticide is dark) or label (labels with dark color) (7/669, 1%); no reason (36/669, 6%).

## Discussion

This study reaffirms that most pesticides used for self-harm are obtained from the house or its close surrounding [13,15]. In addition, the most common reasons for selecting the particular pesticides were easy accessibility and popularity as a suicidal agent in the person's village. These findings are similar to a small study published in the same province which found around 40% of the pesticide poisoning patients cited easy availability as a main reason for choosing pesticide for self-harm [15]. These findings highlight the importance of accessibility and that measures are needed to ensure safe (locked) storage in and around the home. However, as previous studies found that 24 months after the introduction of safe storage devices only 55% of households have still made use of the devices [9], it appears unlikely that safe storage will be an effective sole strategy to reduce deaths from pesticide poisoning. Complementary strategies such as reducing the availability of the most highly toxic pesticides through education and regulation need to be explored along with community intervention to provide alternative ways of dealing with stress or reducing other precipitant such as alcohol ingestion [18] or altering the pesticide formulation to reduce the gastrointestinal absorption of pesticides [19]. Further research on factors such as packaging that could influence the choice of a pesticide for self-harm when it is easily accessible is also important area to be considered.

Our study also shows that about half of non-farmers obtained the pesticides they used for self-harm directly from pesticide outlets, emphasizing the importance of regulating sales practices among both government and private sector. Ninety one patients (14%) obtained pesticides from the sales outlet just few hours prior to self-poisoning; most of them were young males. A number of studies in the Europe and USA suggested that regulating the sales and pack size of pharmaceuticals may have reduced the number of suicides with pharmaceuticals [20-22]. Sri Lanka has effectively regulated the import and use of pesticides in agricultural practice, markedly reducing deaths from self-poisoning [23-25]. The current legislation in Sri Lanka allows for the regulation of sales and marketing and more attention could be given to safe sale practices among the public.

### Strengths and Limitations of the study

The main strength of this study was the large sample size and the ability to collect data soon after self-harm attempt. There are two main limitations. First, we only obtained data from the patients who presented to large referral hospital and this may under-estimate the total number of poisonings in the study area. For example, a number of patients may have died before reaching the hospital, others may have taken small amounts of poison and been managed in peripheral hospitals. Furthermore, we excluded 36 patients from the study as they died soon after admission and a further 213 patients' interviews were incomplete. This may have resulted in a biased assessment of the sources of pesticide used in act of self-harm. However, we didn't find any difference in source of pesticide in participants who died in hospital compared to survivors. Second, we did not investigate the level of intent among these patients; this might have impacted on the way they have accessed the pesticides from a location.

## Conclusion

We found that most people who used pesticides in acts of self-poisoning used chemicals that were already available within the home or in close proximity. This highlights the importance of reducing the accessibility of pesticides in the domestic environment and suggests that it may be possible to reduce deaths from self-poisoning if safe storage practices and proper storage devices are used within farming communities. As it is unlikely that pesticides will always be safely stored, it remains important that the most toxic pesticides continue to be phased out of the market. Further investigation of how the sales of pesticides could be made safer may reduce the number of people with intentions of self harm accessing pesticides from a shop.

## Competing interests

The authors declare that they have no competing interests.

## Authors' contributions

FM involved in collection, processing and analysis of data and wrote the paper, GM and ME are involved in designing the study and revising the manuscript, SA was the visiting physician of the study hospital and supervises the overall recruitment process and helped in data collection, DG and FK involved in statistical analysis and writing the paper and giving guidance to FM on the manuscript writing. AD supervised over all data collection for the study and helped in manuscript writing. All authors contributed to the final version of the manuscript and approve its submission.

## Pre-publication history

The pre-publication history for this paper can be accessed here:


